# Exploring metabolomic dynamics in acute stress disorder: amino acids, lipids, and carbohydrates

**DOI:** 10.3389/fgene.2024.1394630

**Published:** 2024-07-25

**Authors:** Nicholas C. Gary, Burook Misganaw, Rasha Hammamieh, Aarti Gautam

**Affiliations:** ^1^ Medical Readiness Systems Biology, Walter Reed Army Institute of Research, Silver Spring, MD, United States; ^2^ The Geneva Foundation, Tacoma, WA, United States; ^3^ Culmen International, Alexandria, VA, United States

**Keywords:** acute stress disorder (ASD), PTSD, posttraumatic stress disorder, metabolomics (OMICS), biomarker, lipids

## Abstract

Acute Stress Disorder (ASD) is a psychiatric condition that can develop shortly after trauma exposure. Although molecular studies of ASD are only beginning, groups of metabolites have been found to be significantly altered with acute stress phenotypes in various pre-clinical and clinical studies. ASD implicated metabolites include amino acids (β-hydroxybutyrate, glutamate, 5-aminovalerate, kynurenine and aspartate), ketone bodies (β-hydroxybutyrate), lipids (cortisol, palmitoylethanomide, and N-palmitoyl taurine) and carbohydrates (glucose and mannose). Network and pathway analysis with the most prominent metabolites shows that Extracellular signal-regulated kinases and c-AMP response element binding (CREB) protein can be crucial players. After highlighting main recent findings on the role of metabolites in ASD, we will discuss potential future directions and challenges that need to be tackled. Overall, we aim to showcase that metabolomics present a promising opportunity to advance our understanding of ASD pathophysiology as well as the development of novel biomarkers and therapeutic targets.

## 1 Introduction

Acute stress disorder (ASD) is a prevalent psychiatric condition that can develop among trauma exposed individuals (Adler and Gutierrez, 2022; Association, 2013). According to DSM-V classification, ASD is an intense and debilitating reaction occurring in the immediate aftermath of an overwhelming traumatic event persisting for 3 days and lasting less than a month (Adler and Gutierrez, 2022; Ontario Health Quality, 2021). The International Classification of Diseases (ICD-10) criterion describes acute stress reactions (ASR) as a transient response observed immediately post-trauma that normally resolves within 2–3 days. ASD shares a large proportion of its symptomatology with other trauma-related psychiatric disorders such as post-traumatic stress disorders (PTSD), the main distinguishing factor being the timing and duration of the symptoms with respect to the trauma event. It was initially emphasized that a person experiencing ASD should satisfy four symptom cluster such as dissociation, intrusion, avoidance, and arousal and should satisfy the five dissociative symptoms (Bryant et al., 2011; Fanai and Khan, 2023).

ASD is a well-documented precursor for long-term psychiatric sequalae of trauma exposure. Research has found that about 80% of people that met ASD symptom criteria following acute trauma will go on to develop PTSD 6 months later (Shalev, 2009). Still, it should be noted that an ASD diagnosis is not necessarily intended to function as a predictor of subsequent PTSD since at least one-third of people who develop PTSD do not meet DSM-5 ASD criteria (Du et al., 2022; Wakefield, 2015; Schnyder et al., 2015). Other long-term consequences of ASD include mood disorders and substance abuse disorder (Fanai and Khan, 2023).

There are no validated risk factors for ASD, however, considering its similarly to PTSD, pre-trauma, peri-trauma, and post-trauma factors that may be involved in ASD pathophysiology have been described (Fanai and Khan, 2023). The probability of ASD onset depends on the type and severity of the trauma. For example, 33% of people who experienced a mass shooting develop ASD, while 14% of people who experienced a traumatic brain injury developed ASD (The Recovery Village and Hill, 2023). Other factors such as past psychiatric diagnosis, prior trauma, genetics, and being female increases risk for developing ASD.

During the acute stress phase, the body undergoes profound metabolic alterations leading to changes in metabolite abundances in the brain, peripheral tissue, and the subcellular level (Morella et al., 2022; Picard and McEwen, 2018; Yoganathan et al., 2023; Rabasa and Dickson, 2024). Current research understands that acute psychosocial stress has numerous effects on human cognition, however, the role of specific metabolic dysregulation that occurs in the brain during ASD is not well understood. (Spencer et al., 2024). In this review, we summarize metabolites, biomolecules of smaller molecular weight, that have been reported to be associated with ASD. Our goal in bringing attention to specific metabolites is to start drawing associations with metabolic pathways that could be potentially dysregulated during ASD. Deciphering specific metabolic changes can enable development of prognostic biomarkers and intervention strategies to mitigate the impact of ASD. Towards this end, there are recent efforts to study the relevance of various metabolites to ASD onset and progression. We discuss four categories of metabolites: amino acids, ketone bodies, lipids, and carbohydrates. To further understand the dynamic interplay of these metabolites that are individually associated with ASD, we will also explore physiological and functional themes by performing pathway and network analysis.

## 2 Metabolites associated with acute stress phenotypes

Although metabolomics study of acute stress related phenotypes began recently, given the central role of small molecules for stress response variabilities, there have been a few studies and promising findings. To find articles discussing ASD and metabolomics, we used the PubMed database for our literature search. Specifically, our keyword searches were a combination of “Acute Stress Disorder,” or “Acute Stress Response,” or “Acute Psychological Stress,” and “Metabolomics,” or “Metabolites.” By compiling results from literature search of clinical and pre-clinical studies, we want to highlight the most current research-based evidence. These include both preclinical studies with various animal models as well as clinical studies of military and civilian cohorts. We excluded pre-clinical studies that did not use rodent or NHP models. Most metabolites that have been reported to be associated with ASD can be grouped into four broad categories: 1) amino acids, 2) ketone bodies 3) Lipids, and 4) carbohydrates.

### 2.1 Amino acids

Amino acids (AA) are the building blocks of proteins (Shen and Sergi, 2024) and are involved in repair mechanisms, cellular signaling, and energy production (Tipton and Wolfe, 2001). Specific metabolites belonging to certain classes of non-essential AA (AA that can be synthesized by the body) are affected by stress exposure and are significantly associated with acute stress phenotypes (Morgan et al., 2022; Son et al., 2021; Puchalska and Crawford, 2017; Nehlig, 2004). A study using saliva samples from a military population before, during, and after a 72-h mock mission identified 30 significant metabolomic pathways during the acute stress episode (McKetney et al., 2022). Notably, aspartate and glutamate involved in energy breakdown process were identified to be significantly increased. Elevated levels of blood glutamate was found in another study on 229 post-traumatic injury patients (Li et al., 2023) where longitudinal sample were collected 1-, 3-, 4-, and 28-days after hospital admission.

Increased Glutamate release within the limbic and cortical brain regions may be a result of glucocorticoid release post-stress (Lowy et al., 1993; Lowy et al., 1995). Within the brain, glutamate acts on various receptors, including N-methyl-D-aspartate (NMDA) receptors and α-amino-3-hydroxy-5-methyl-4-isoxazolepropionic acid (AMPA) receptors (Piepoli et al., 2009). Activation of these receptors contributes to synaptic plasticity, which is vital for learning and memory processes (Pal, 2021). The heightened release of glutamate contributes to excitatory signaling, influencing neuronal activity and communication (Petroff, 2002). This excitatory neurotransmission is closely linked to the activation of the hypothalamic-pituitary-adrenal (HPA) axis, a key component of the body’s stress response system which then triggers the release of stress hormones, such as cortisol, further amplifying the overall stress response (Wadsworth et al., 2019; Leistner and Menke, 2020; Lee et al., 2022; Kinlein et al., 2019). Glutamatergic signaling is also found to be intricately involved in the regulation of mood and emotional responses (Yuan and Hou, 2015).

The involvement of kynurenine pathway in ASD have also been documented which is the major route of degradation of the essential amino acid tryptophan, accounting for ∼95% of dietary tryptophan disposal. Concentrations of Kynurenine were significantly lower after stress induction compared to pre-stress induction in a study that used Maastricht Acute Stress Test (MAST) (Shilton et al., 2017) in a cohort of 56 male participants (La Torre et al., 2021). Stress-induced immune activation stimulates the production of pro-inflammatory cytokines leading to activation of kynurenine pathway (Dhabhar, 2014; Mingoti et al., 2023; Deng et al., 2021). Elevated kynurenine has been associated with neuroinflammation and altered neurotransmission where the amino acid tryptophan, have a crucial role in depression (Michels et al., 2018; Kim and Jeon, 2018; Mithaiwala et al., 2021; Li et al., 2021). This immune-neuro crosstalk suggests that kynurenine serves as a signaling molecule connecting peripheral immune activation to central nervous system alterations during stress (Herhaus et al., 2021; Dostal et al., 2017; Vecchiarelli et al., 2016).

### 2.2 Ketone bodies

Acute stress can trigger the release of stress hormones that affect production of ketone bodies, which are metabolized from free-fatty acids in the liver and used by the brain in the event of energy starving states (Evans et al., 2017). A mouse study modeling acute stress using an immobilization stressor found significantly increased levels of Beta-Hydroxybutyrate (BHB) in the prefrontal cortex and blood (Son et al., 2021). BHB becomes dysregulated in ASD and may affect the body’s ability to supply nutrients for healthy function and thereby impacting brain energy metabolism and overall neuronal health. BHB has been shown to enhance the effects of gamma-aminobutyric acid (GABA), an inhibitory neurotransmitter that regulates neuronal excitability. This GABAergic modulation may contribute to the calming effects observed in stressful situations and appears to have anti-inflammatory properties, impacting immune responses associated with stress. By inhibiting inflammatory pathways, BHB may mitigate the detrimental effects of chronic stress on the body. This anti-inflammatory action by BHB activation is thought to occur through the inhibition of NLRP3 inflammasome activation, a key component of the innate immune system (Youm et al., 2015). Furthermore, BHB is known to activate specific cellular signaling pathways, such as the Nrf2 antioxidant pathway. This activation may confer protection against oxidative stress, which is often heightened during acute stress episodes (Janšáková et al., 2021). By enhancing cellular resilience, BHB could potentially mitigate the negative impact of stress on the body’s tissues (Izuta et al., 2018).

Overall, BHB has emerged as a multifaceted molecule with potential implications in stress modulation. Its influence on GABAergic neurotransmission, anti-inflammatory properties, and activation of cellular protective pathways suggests that BHB may play a role in mitigating the physiological effects of acute stress (Yamanashi et al., 2020).

### 2.3 Lipids

Cortisol and adrenocorticotropic hormone (ACTH), are secreted in response to stress and plays a central role in the hypothalamic-pituitary-adrenal axis (HPA), the primary stress response mechanism of mammals (Hannibal and Bishop, 2014; Pulopulos et al., 2020; Lightman et al., 2020; Rivier and Vale, 1983). Cortisol secretion is activated by ACTH which, in turn, increases blood pressure and dilates pupils, both of which are found in association with increased cardiovascular disease risk (John-Henderson et al., 2020; Langer et al., 2022). Studies have shown other steroid hormones such as testosterone to also be affected in response to acute stress (Sze and Brunton, 2020; Rostamkhani et al., 2012; Kelly et al., 2022). This hormone surge prompts the mobilization of triglycerides from adipose tissue providing a rapid source of energy (Du et al., 2016; Maduka et al., 2015). The breakdown of triglycerides into fatty acids enables cells and tissues to meet the high energy demand during stress response. Significantly lower saliva cortisol awakening response levels were found in association with ASD in 51 subjects in the first few hours after verbal or physical trauma (Marin et al., 2019). ACTH along with cortisol is also found to be significantly increased in association with acute stress in blood samples from 19 patients who underwent a Trier Social Stress Test (TSST) (Lennartsson et al., 2015). Acute mental stress also found significant association with hormonal increase in norepinephrine, as well as other dopaminergic neurons indicating the potential effects on cognition during periods of stress (Bloomfield et al., 2019; Musazzi et al., 2019; Motta e Motta et al., 2021).

The endocannabinoid system that includes molecules like palmitoylethanomide (PEA) and N-palmitoyl taurine plays an important role in regulating stress response. A global metabolic analysis identified increased levels of PEA and N-palmitoyl taurine in association with response to a water avoidance stress mouse model (Lee et al., 2023). PEA is an endogenous fatty acid mediator that is synthetized from membrane phospholipids by N-acyl phosphatidylethanolamine phospholipase D and is linked to inflammation modulation due to endocannabinoid dysregulation (Gugliandolo et al., 2017). Interestingly, the endocannabinoid system and PEA specifically has been implicated in diminishing inflammation found in the brain due to anxiety (Lama et al., 2022).

Overall, lipids are integral part of the physiological adaptation to acute stress. Their role in energy related pathways, membrane structure and synthesis of signaling molecules place them as essential contributors in ASD. Multiple studies show hormonal surge post-ASD can potentially be implicated in lipid metabolism dysregulation leading to future physiological consequences (Rabasa et al., 2019; Motta e Motta et al., 2021; Dlugos et al., 2012).

### 2.4 Carbohydrates

During stress, carbohydrates stored as glycogen in the liver and muscles, and circulating in the bloodstream as glucose, are rapidly broken down to meet the increased energy demands. (Roh et al., 2016). Elevation in glucose level provides for the quick energy demand as a part of acute stress response; however, persistent elevation of blood glucose can contribute to development of metabolic disorders such as diabetes (Lee et al., 2023; Levin et al., 1999; Kilcoyne and Joshi, 2007). A decreased level of glucose was found in blood and saliva from male soldiers immediately pre- and post-submersion into cold water (Kelly et al., 2022). An increased level of glucose and insulin levels was observed in blood in male soldiers after acute psychosocial stress (Nowotny et al., 2010). In general, dysregulation in the levels of glucose signals that the body is under stress (Sancini et al., 2017). Mannose, a C-2 epimer of glucose (Lee et al., 2023), was significantly increased during an acute stress response (Lee et al., 2023). However, increase in glucose may not necessarily be a result of the body directly impacted by Acute Stress and could be due to a delay in metabolite breakdown or dysregulation that happened earlier in the pathway, so these results should not be interpreted as causal.

### 2.5 Relationship among the ASD associated metabolites

**FIGURE 1:**
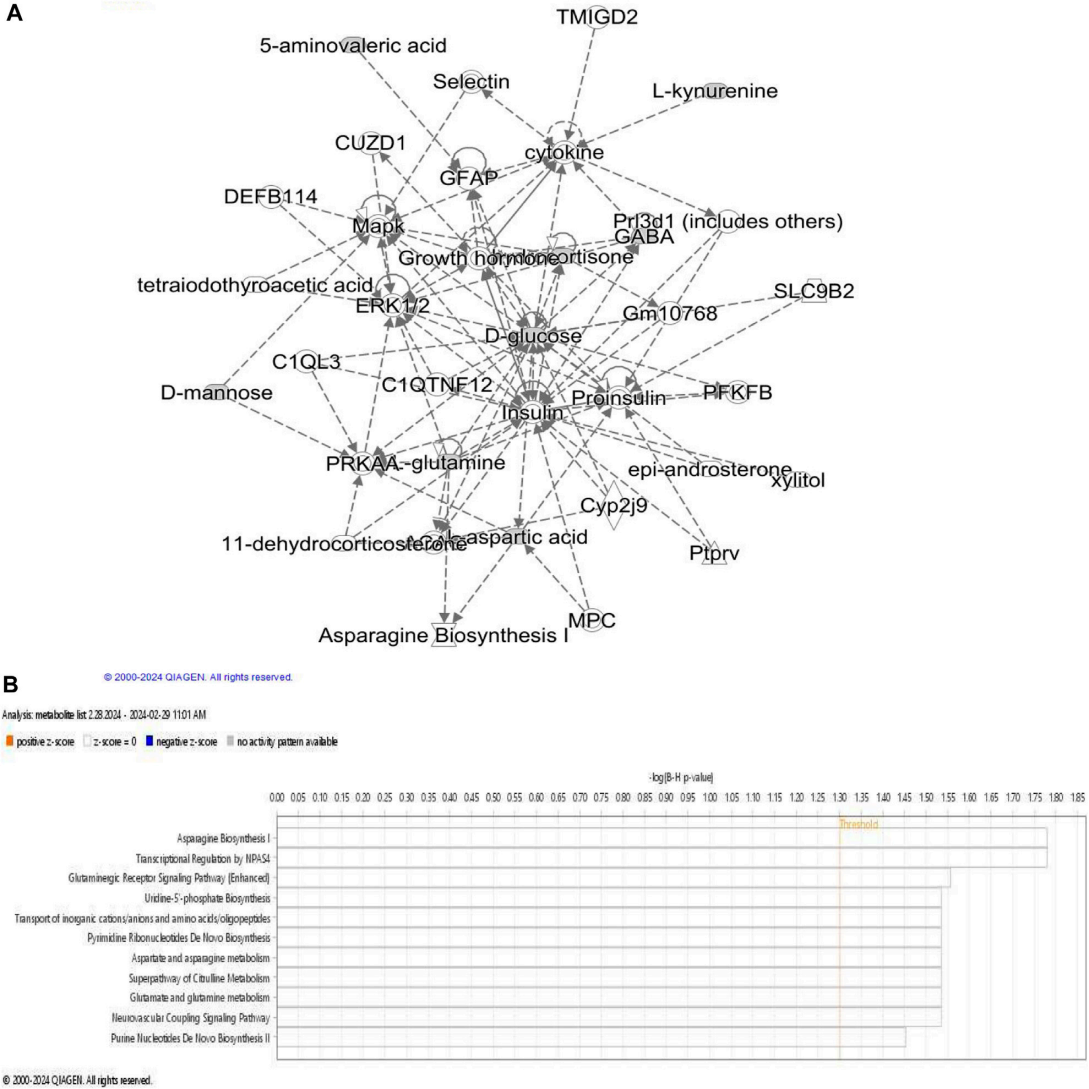
Metabolites associated with acute stress. **(A)** Network describing metabolic disease, hereditary and developmental disorder is obtained using IPA network analysis of ASD associated metabolites. ERK1/2 appears at the center of the network; LEP levels affects glutamine, glutamate, insulin, palmitic acid, pro-insulin, LDL, CAMK2A, NFKB, ACTH and JNK which are also visible in this network. Dashed line represents indirect relationship. **(B)** Overview of pathways significantly enriched in the set of ASD relevant metabolites.

To provide biological context to the shortlisted ASD relevant metabolites (Table 1), we sought to investigate shared, if any, biological themes and categories they represent using IPA (Ingenuity pathway analysis) knowledge database. Metabolites were mapped to canonical pathways using their unique identification numbers (KEGG or PubChem IDs) and a network of metabolites is generated (Figures 1B,C).

**TABLE 1:** Metabolites associated with ASD.

Metabolite	Class	Pathway	References	Study setting	Findings
Glutamate	Amino Acid	Glutamate metabolism	Li et al. (2023)	52 ASD-positive and 177 ASD-negative participants are included. ASD status was evaluated using acute stress disorder scale (ASDS)	Glutamate is increased in serum of ASD diagnosed patients
5-aminovalerate	Amino Acid	Lysine metabolism	Lee et al. (2023a)	Acute stress response was modeled using water avoidance stress (WAS) in 8–10 weeks old male mice (n = 6)	5-aminovalerate level is significantly increased in the brain of acute WAS mice compared to normal controls
Glutamine	Amino Acid	Glutamate Metabolism	Son et al. (2021), McKetney et al. (2022)	male mice exposed to immobilization stress and biospecimen (blood and brain) are obtained 30 min after restraint Saliva samples (n = 30) collected before, during and after an acute stress training event	Glutamate pathway was enriched post trauma
Kynurenine	Amino Acid	Tryptophan Metabolism	La Torre et al. (2021)	Male subjects (n = 56) blood samples	KYN pathway metabolites were lower after acute psychosocial stress
Aspartate	Amino Acid	Alanine and Aspartate Metabolism	McKetney et al. (2022)	Saliva samples (n = 30) collected before, during and after an acute stress training event	Aspartate pathway was enriched post trauma
β-hydroxybutyrate (BHB)	Ketone Body	Glutathione metabolism	Son et al. (2021)	Male mice exposed to immobilization stress and biospecimen (blood and brain) are obtained 30 min after restraint	BHB is increased in prefrontal cortex and blood
GABA	Lipid	Neurotransmitter	Houtepen et al. (2017)	Healthy adult males (n = 29) were exposed to acute psychosocial stress (Trier Social Stress Test) and *in vivo* medial prefrontal GABA level is measured using magnetic resonance spectroscopy	No significant relationship is detected
Cortisol	Lipid	Corticosteroids	La Torre et al. (2021)	Healthy adult male (n = 56) exposed to Maastricht Acute Stress Test	Cortisol level is elevated in saliva in the first few minutes after stress exposure
Glucose	Carbohydrate		Nowotny et al. (2010)	Acute psychosocial stress was measured using trauma script exposure in fifteen (n = 15) male war refugees	Glucose level is significantly increased after exposure to stress
Mannose	Carbohydrate		Lee et al. (2023a)	Acute stress response was modeled using water avoidance stress (WAS) in 8–10 weeks old male mice (n = 6)	Mannose level is significantly increased in mice brain tissue compared to controls
Adrenocorticotropic hormone (ACTH)	Polypeptide Hormone	hypothalamic-pituitary-adrenal axis role	Lennartsson et al. (2015)	Patients with clinical burnout (n = 19) and healthy controls (n = 37) underwent Trier Social Stress Test	Plasma ACTH increased significantly following stress exposure, patients with severe symptoms showing a slightly blunted response

Further examination of metabolite specific networks identified a network consisting of 35 biomolecules associated with diseases and biofunctions “amino acid metabolism, endocrine system development and post-translational modifications.” Extracellular signal-regulated kinases (ERK1 and ERK2) were observed in this network which has not been directly associated with ASD in previous studies. ERK 1 and ERK 2 are evolutionarily conserved serine-threonine kinases that are involved in regulating cell signaling and Erk pathway can activate c-AMP response element (CRE) binding (CREB) protein via glutamic acid and calcium dependent protein kinase (CAMK2A). Interestingly, CREB signaling pathway has been shown to be associated with PTSD diagnosis (Manners et al., 2019). GABA association with glucose was found, which has been implicated in sleep disturbances (Tsuneki et al., 2016). ERK is also involved in cognition and emotional behavior during adulthood (Albert-Gascó et al., 2020). Given that it has important implications in various neurodegenerative diseases, it is crucial to assess its relevance in ASD pathophysiology and its potential role in linking ASD and PTSD.

### 2.6 Relevance of metabolites to other stress-related psychiatric disorders

It is interesting to note that the aforementioned sets of ASD relevant metabolites are largely overlapping with those associated with other relatively well-studied psychiatric illnesses (Mellon et al., 2018; Nedic Erjavec et al., 2017; Bryant, 2011). Amino acids have a crucial role in neurotransmitter synthesis and glutamate disruption, leading to lower concentrations of the amino acid in the brain, which have been linked to major depressive disorder (MDD) (Nasir et al., 2020). Similar to ASD, Kynurenine pathway was affected with a decrease in concentrations of tryptophan and kynurenine (Almulla et al., 2022; Ormstad et al., 2016; Sa et al., 2012; Lin et al., 2023). Imbalances in glutamate levels have also been linked to various neuropsychiatric disorders, including anxiety and depression, highlighting the significance of understanding the glutamate-mediated mechanisms in ASD pathophysiology (Graybeal et al., 2012).

Not unlike ASD, chronic inflammation has been associated with depression and PTSD (Rohleder, 2019; Lee and Giuliani, 2019; Hori and Kim, 2019; Quinones et al., 2020). Lipids have been implicated as metabolites significantly altered in individuals diagnosed with PTSD (Huguenard et al., 2020; Bharti et al., 2022; Konjevod et al., 2022). Persistent elevation of lipids in PTSD patients have been shown to lead to hyperlipidemia (Jergović et al., 2015; Nkiliza et al., 2023). Other stress-related psychiatric conditions such as MDD is also linked with a change in blood lipids (Zhang et al., 2022).

The relationship between carbohydrates and serotonin, neurotransmitters involved in mood regulation, is noteworthy. Carbohydrate consumption can lead to increased serotonin production, potentially influencing mood, and emotional wellbeing (Wurtman and Wurtman, 1996; Wurtman, 1984). This connection suggests that the intake of carbohydrates may have a role in modulating the psychological aspects of the stress response.

## 3 Conclusions and perspectives

Accumulating evidence from recent studies has indicated the crucial role of metabolic regulation for ASD pathophysiology. Although the study of metabolomics in acute stress variables is still relatively young, some notable insights has already been revealed. In order to accelerate and optimize the impact of these findings, future studies should focus on addressing challenges surrounding sample collection for large-scale human trails. First, most existing studies are preclinical investigations utilizing various animal/experimental models. Although metabolites are largely conserved across species, some of the preclinical findings may not necessarily readily translate to humans. A potential shortcoming of psychiatric studies on animals is that there is no concrete way to diagnose a psychiatric condition, since human diagnoses often rely on questionnaires, survey, and scores that could not be implemented on non-human models. Thus, large-scale human studies are critical for filling this (translational) gap.

Second, existing studies are limited to elucidating a handful of targeted metabolites preselected based on a hypothesis driven by findings in other psychiatric conditions. Recent technological advances in liquid chromatography-mass spectrometry (LC/MS) enabled accurate measurements/quantification of high-throughput untargeted metabolomics which can detect a large number of metabolites simultaneously (Gertsman and Barshop, 2018). ASD studies that employ such high-throughput approaches should be prioritized.

Third, metabolite abundances are significantly influenced by several potential confounding factors, affecting specificity and reproducibility of study findings. Variables such as preexisting psychiatric and metabolic conditions, type and severity of trauma, comorbidities (TBI, physical injury), ASD assessment method as well as sociodemographic factors need to be carefully considered. Furthermore, existing studies comprise modest sample size cohorts. Larger adequately powered studies are needed to identify reproducible findings and thoroughly characterize the role and interplay of metabolomics and ASD outcomes.

Lastly, beyond a mere association test, delineating the direction of causal influences (i.e., metabolic alterations that predispose to ASD susceptibility from those that are consequences of ASD onset) is crucial for real-world application purposes. Future studies should leverage modern bioinformatics and machine learning techniques. Longitudinal studies that can map time profiles of metabolite trajectories (distinguish transitory responses from persistent longer-duration maladaptive changes) should be conducted.

The potential benefit from metabolic studies is profound and wide-ranging; from developing biomarkers to identifying therapeutic targets. ASD is an important precursor for subsequent psychiatric illnesses in the longer term. Metabolomic events are downstream molecular consequences closer to the physiological changes and are likely to have a larger effect size and more readily interpretable results.

Future studies that further interrogate metabolomics with stress exposure and its neuropsychiatric sequalae will allow development of prognostic biomarkers and novel preventative strategies. Overall, metabolomics studies present a unique and promising opportunity for understanding the molecular underpinnings of ASD onset and progression, thereby providing new avenues for development of biomarkers and therapeutic targets.
